# Transcriptomic Characterization of Genes Harboring Markers Linked to Maize Yield

**DOI:** 10.3390/genes15121558

**Published:** 2024-11-29

**Authors:** Agnieszka Tomkowiak, Tomasz Jamruszka, Jan Bocianowski, Aleksandra Sobiech, Karolina Jarzyniak, Maciej Lenort, Sylwia Mikołajczyk, Monika Żurek

**Affiliations:** 1 Department of Genetics and Plant Breeding, Poznań University of Life Sciences, Dojazd 11, 60-632 Poznań, Poland; aleksandra.sobiech@up.poznan.pl (A.S.); maciej.lenort@up.poznan.pl (M.L.); sylwia.mikolajczyk@up.poznan.pl (S.M.); 2 Department of Mathematical and Statistical Methods, Poznań University of Life Sciences, Wojska Polskiego 28, 60-637 Poznań, Poland; jan.bocianowski@up.poznan.pl; 3 Department of Biochemistry and Biotechnology, Poznań University of Life Sciences, Dojazd 11, 60-632 Poznań, Poland; karolina.jarzyniak@up.poznan.pl; 4 Plant Breeding and Acclimatization Institute—National Research Institute, Radzików, 05-870 Błonie, Poland; m.zurek@ihar.edu.pl

**Keywords:** next-generation sequencing, candidate genes, PCR, qPCR, yield, maize

## Abstract

Background: It is currently believed that breeding priorities, including maize breeding, should focus on introducing varieties with greater utility value, specifically higher yields, into production. Global modern maize breeding relies on various molecular genetics techniques. Using the above mentioned technologies, we can identify regions of the genome that are associated with various phenotypic traits, including yield, which is of fundamental importance for understanding and manipulating these regions. Objectives: The aim of the study was to analyze the expression of candidate genes associated with maize yield. To better understand the function of the analyzed genes in increasing maize yield, their expression in different organs and tissues was also assessed using publicly available transcriptome data. Methods: RT-qPCR analyses were performed using iTaq Universal SYBR Green Supermix (Bio-Rad, Hercules, CA, USA) and CFX96 Touch Real-Time PCR Detection System (Bio-Rad, Hercules, CA, USA). Each of the performed RT-qPCR experiments consisted of three biological replicates and three technical replicates, the results of which were averaged. Results: The research results allowed us to select three out of six candidate genes (*cinnamoyl-CoA reductase 1—CCR1*, *aspartate aminotransferase—AAT* and *sucrose transporter 1—SUT1*), which can significantly affect grain yield in maize. Not only our studies but also literature reports clearly indicate the participation of *CCR1*, *AAT* and *SUT1* in the formation of yield. Identified molecular markers located within these genes can be used in breeding programs to select high yielding maize genotypes.

## 1. Introduction

Maize (*Zea mays* L.) is one of the most important and oldest crop species. Teosinte (*Z. mays* ssp. pavglumis) is considered to be an ancestor of maize. Genetic loci such as *tb1* (*teosinte branched* 1) and *tga1* (*teosinte glume architecture 1*) played a key role in the evolution of teosinte into modern maize [1,2,3]. The cultivation range of maize is wide, between 50° N and 40° S. Currently, grain maize is grown on about 197 million ha of land worldwide, making it the second most important economic crop after wheat. Annual grain production of maize in the world currently amounts to 1137 million tons, and maize yields have increased by almost 2 tons over 25 years (from 3.9 to 5.8 tons/ha). The above-mentioned intensive increase in maize yields would not have been possible without biological progress.

This progress can be described as an ecological method for intensifying agricultural production, involving the genetic improvement of plants to become more efficient in utilizing natural resources and industrial means of production [4,5]. The search for new genes that are important from the economic point of view is a task for modern plant breeding. Nowadays, maize breeding programs prioritize the introduction of varieties with increased utility value, i.e., higher yield and improved nutritional, feed, and technological quality of the obtained yield [6].

Modern maize breeding globally relies on a diverse array of research techniques in the field of molecular genetics [7,8]. These technologies enable us to identify genomic regions associated with various phenotypic traits, including yield. Understanding their molecular determinants is crucial to manipulating them. The application of genomic methods in the improvement of crop species, including maize, can effectively contribute to solving problems related to food shortages. In the last two decades, many scientists [9,10,11,12,13] have used molecular biology methods in their studies to detect and localize loci determining grain yield and yield structure traits in maize. Identification of the locus associated with yield and characterization of candidate genes located in it can influence the evolution of agriculture to meet the global demand for both food supplies and new biomaterials.

The next step will be to use molecular markers linked to candidate genes in breeding programs to select high-yielding genotypes. The use of Marker-Assisted Selection (MAS) will allow us to shorten the breeding process. Molecular markers are widely utilized in breeding programs, particularly for selecting genotypes with economically important traits [14,15,16,17,18]. Molecular markers have been successfully used in maize breeding for many years [19,20].

Another important problem in maize breeding is related to assessing the genetic purity of parental inbred lines of maize. This is an essential quality control function in maize hybrid breeding [21]. These functions are now more critical due to the stringent intellectual property requirements governing plant breeding and variety registration in many countries [22]. Inbred lines’ genetic purity and parentage can be proved using two approaches, namely, the use of biochemical markers and use of molecular markers. Several types of molecular markers are available for the detection of polymorphisms [23]. The main ones include simple sequence repeats (SSRs) and single-nucleotide polymorphisms (SNPs). Recent advances in molecular technology have emphasized the use of SNP markers because they are cost-effective per data point, provide locus specificity, are codominant, have adequate genomic abundance, and have the potential for high throughput, unlike the other markers [24]. The application of molecular markers is more efficient and saves time and resources [25].

The aim of our study was to develop primers and methods for identifying selected molecular markers located within candidate genes determining the yield of maize grain. These markers could be used to select high-yielding genotypes. This publication also analyzed the expression level of candidate genes to confirm their participation in yield formation. In order to better understand the function of the analyzed genes in increasing maize yield, their expression in various organs and tissues was also assessed using publicly available transcriptome data.

## 2. Materials and Methods

### 2.1. Material

The plant material used for the study consisted of 10 maize genotypes (5 high-yielding genotypes—SP6, KP12, KP13, SP8 and KP15—and 5 low-yielding genotypes—Blask maternal form, Blask paternal form, UP10, UP20 and UP30). The genotypes came from the collection located at the Department of Genetics and Plant Breeding of the Poznań University of Life Sciences and Polish breeding companies: Małopolska Plant Breeding Co., Ltd. (Kraków, Poland) and Plant Breeding Smolice Co., Ltd. IHAR Group (Warszawa, Poland).

### 2.2. Methods

#### 2.2.1. Field Experiment

The field experiment was established on 10 m^2^ plots, in a randomized complete block design, in three replications on the premises belonging to Małopolska Plant Breeding Co. Ltd. in Kobierzyce, Poland (50°58′17″ N, 16°55′50″ E).

#### 2.2.2. Weather Conditions in the Area Where the Field Experiment Was Established

The average temperature in the area where the field experiment was established was 9.5 °C in 2022, which means that it was 0.7 °C warmer than the temperature in the multi-year period 1991–2020. The average amount of precipitation in this area was 495.3 mm. Analysis of climate conditions in 2023 showed that the average annual temperature in the area where the field experiment was established was 1.3 °C higher than the temperature in the years 1991–2020, making 2023 one of the warmest years on record. The average amount of precipitation in this area was 652.1 mm, which exceeded the average precipitation levels from 2022. In 2024, from January to the end of September, the average temperature was 1.6 °C higher than the levels recorded from 1991 to 2020, and the average precipitation reached 672.1 mm. This suggests that 2024 may be even warmer than 2023.

#### 2.2.3. Selection of Candidate Genes and Their Associated Molecular Markers

Candidate genes were selected based on the results previously published by Tomkowiak et al. [12], who used next-generation sequencing, association mapping and physical mapping to identify candidate genes related to yield. In this publication, we studied the expression of these genes in high- and low-yielding genotypes to confirm their effect on yield. We also checked whether specific molecular markers located within these candidate genes differentiate high- and low-yielding genotypes and whether they can be used for selection in breeding programs. The selected markers and their linked genes are given in Table 1.

#### 2.2.4. Total RNA Isolation and Reverse Transcription Reaction

Total RNA isolation from young maize seedlings was performed using the Maxwell RSC Plant RNA isolation kit (Promega, Madison, WA, USA). The concentration and purity of isolated total RNA were measured using a NanoDrop. cDNA synthesis was performed using 1 μg of RNA using the iScript Reverse Transcription Supermix for RT-qPCR kit (Bio-Rad, Hercules, CA, USA) according to the manufacturer’s protocol. The temperature profile of the cDNA synthesis reaction was as follows: preincubation for 5 min at 25 °C, reverse transcription for 60 min at 46 °C, reverse transcriptase inactivation for 1 min at 95 °C, cooling to 4 °C, and storage at −20 °C.

#### 2.2.5. qPCR Analyses

Sequences of the candidate genes were found in the BLAST database and downloaded in FASTA format. The sequences were used to design primers for RT-qPCRs using Primer3Plus (Table 2).

RT-qPCR analyses were performed using iTaq Universal SYBR Green Supermix (Bio-Rad, Hercules, CA, USA) and CFX96 Touch Real-Time PCR Detection System (Bio-Rad, Hercules, CA, USA). Each of the performed RT-qPCR experiments consisted of three biological replicates and three technical replicates, the results of which were averaged. Simultaneously, for each tested gene, negative control without cDNA template—NTC (No-Template Control) was performed in three technical replicates. The composition of the RT-qPCR mixture was as follows: iTaq supermix—5 μL; forward and reverse primers (10 μM)—0.5 μL each; 3 μL of nuclease-free water; and cDNA template—1 μL. The following temperature profile was used in RT-qPCRs: initial denaturation for 3 min at 95 °C, followed by 40 cycles; denaturation for 10 s at 95 °C; and finally, annealing for 30 s at 53.5 °C. During the melting stage (melting curve), the temperature ranged from 65 °C to 90 °C; every 5 s, the temperature was increased by 0.5 °C. Expression analysis for this experiment was performed using Bio-Rad CFX Maestro software v. 2.3 and the GeneStudy tool (Bio-Rad, Hercules, CA, USA), obtaining normalized gene expression values. The expression results of the studied genes were related to the expression values of reference genes, which have a stable expression profile for a given experiment. This allowed normalized expression values of the studied genes to be obtained. Based on the literature data, two reference genes were selected: *β*-tubulin (*β* TUB) and cyclophilin (CYP) [21].

#### 2.2.6. Transcriptomic Data Analysis

Accession numbers used in the analysis of transcriptomic data were found using the National Center for Biotechnology Information (NCBI) website (https://www.ncbi.nlm.nih.gov/, accessed on 6 May 2024). Transcriptomic data were used to quantify the expression levels of identified genes in various *Zea mays* organs and tissues. In the latter study, expression levels were measured in Fragments Per Kilobase of transcript per Million mapped reads (FPKM).

#### 2.2.7. DNA Isolation

To confirm the presence of markers determining high yield in the tested genotypes, genomic DNA was isolated and a PCR was performed. DNA was isolated from the leaves of 10-day-old seedlings using a GeneMATRIX Plant and Fungi DNA Purification Kit (EURx Ltd., Gdańsk, Poland), according to the procedure recommended by the manufacturer. DNA concentration and quality were determined using a DeNovix spectrophotometer (DeNovix Inc., Wilmington, DE, USA). Samples were diluted with buffer (EURx Ltd., Gdańsk, Poland) to obtain a uniform concentration of 30 ng/μL.

#### 2.2.8. PCR Conditions

PCR primers were designed based on analyzed marker sequences. PCR was conducted in a T1000 thermocycler (Bio-Rad, Hercules, CA, USA). The 20 μL reaction mixture contained 5− Green GoTaq Flexi Reaction Buffer, 4 μL; 25 mM MgCl_2_, 1.6 μL; 10 mM Ultrapure dNTPs Mix, 0.32 μL; GoTaq G2 Flexi DNA Polymerase (Promega, Madison, WA, USA), 0.17 μL; nuclease-free water, 10.91 μL; 10 μM forward (F) primer, 0.5 μL; 10 μM reverse (R) primer, 0.5 μL (Table 3); and 50 ng DNA, 2 μL. The PCR conditions were as follows: initial denaturation, 94 °C/2 min; denaturation, 95 °C/1 min; annealing, Ta/50 s (Table 3); primer extension, 72 °C/1 min; final denaturation, 72 °C/5 min; and hold 4 °C/∞, with a total of 40 cycles.

#### 2.2.9. Electrophoresis

Electrophoresis of PCR products was carried out in a 2% agarose gel for 1.5 h at 110 V. First, 5 μL of Midori Green Advance DNA stain (NIPPON, Düren, Germany) solution was added per 100 mL of gel for DNA visualization. PCR product size was assessed using a 50 bp and 100 bp DNA Step Ladder (Promega, Madison, WA, USA). Visualization of separated DNA fragments in the gel by UV light was performed using the Gel Doc XR+ gel documentation system (Bio-Rad, Hercules, CA, USA).

#### 2.2.10. Statistical Analysis

The compatibility of the empirical yield distribution to the normal distribution was verified using the Shapiro–Wilk test [26]. This test assesses the differences between theoretical values of the normal distribution and the empirical sample distribution. A twoway (genotypes and years) analysis of variance (ANOVA) was conducted to examine the effects of genotype and experimental years, as well as their interaction, on maize yield performance. Additionally, a two-way ANOVA was applied to assess the effects of predefined high- and low-yielding genotype groups, years, and their interaction on yield. Mean values and standard deviations were calculated. Subsequently, the least significant difference (LSD), at the 0.05 level, was calculated and used in post hoc analysis to establish homogeneous groups. The interdependence of genotype yields across the experimental years was evaluated based on Pearson’s linear correlation coefficients. A one-way ANOVA was performed to assess the impact of genotypes on normalized gene expression for six genes: *CCR1*, *ArabidopsisWAT1*, *EIF3C*, *KELP_0*, *AAT*, and *SUT1*. For each gene, mean values, standard deviations, and LSD values (at the 0.05 significance level) were computed. The normalized gene expression relationships between genes were estimated using Pearson’s linear correlation coefficients, and the results of these interdependencies were presented via a heatmap. The impact of normalized gene expression for individual genes on the yield of the studied genotypes was evaluated using regression analysis [27]. Dependencies were assessed independently for each year using different regression models [28] and analyzed separately for the six genes. The data were also subjected to multivariate statistical analyses, including canonical variable analysis [29] and Mahalanobis distance [29,30,31]. These analyses were conducted independently for two datasets: (1) phenotypic yield observations over three years and (2) normalized gene expression observations for the six genes. All analyses were performed using the Genstat v. 23.1 statistical software package [32].

## 3. Results

### 3.1. Phenotypic Variability

The maize yield was characterized by a normal distribution. The results of a two-way ANOVA showed that genotypes and years were significant differentiating factors for maize yield (Table 4). On the contrary, genotype–year interaction had no effect on yield (Table 4).

**Figure 1:**
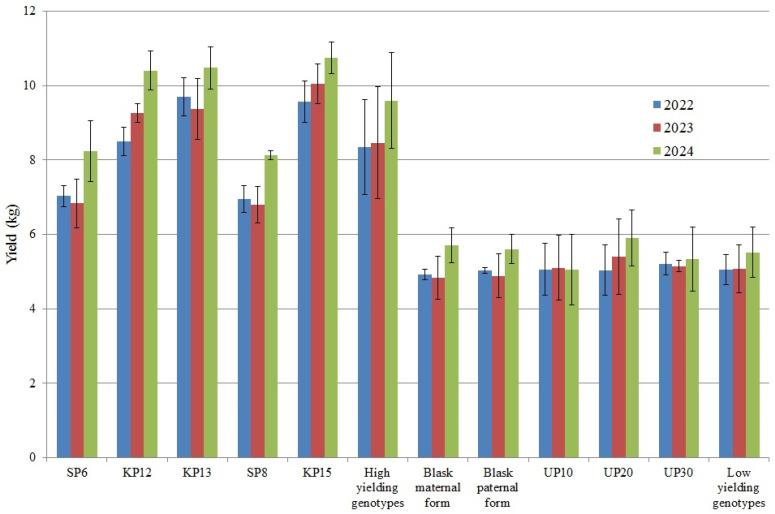
Average yield values of the tested genotypes and groups of high- and low-yielding genotypes in the individual years of the experiment.

The yields of the tested genotypes in the individual years of the experiment are presented in Figure 1 and Table 5. The graph shows that both high and low-yielding genotypes were characterized by the highest yield in 2024. In an analysis of the weather in 2024, the highest average temperature and rainfall were recorded among the years presented. Maize is a thermophilic plant; as a C4 plant, it needs higher air temperatures than, for example, wheat or rapeseed, as well as large amounts of light, which it can use effectively, e.g., by showing greater growth intensity than wheat. For the germination of grains, corn needs a soil temperature of at least 8–10 °C. The coldest year, with the least rainfall, turned out to be 2022, which is why the lowest yields were recorded this year in both groups of genotypes (low- and high-yielding).

The analysis of the interdependence of genotype yields in individual years showed a statistically significant (at the level of 0.001) correlation for each of the three compared pairs. The correlation coefficients between the years 2022–2023, 2022–2024 and 2023–2024 were, respectively, 0.946, 0.925 and 0.938. The results of the conducted ANOVA confirmed statistically significant differentiation of the average yield values of genotype groups designated a priori as high- and low-yielding (Table 6). Significant differentiation was observed in each year of the study and for the averages across years (Table 5). The genotypes classified as high-yielding were characterized by an average yield of 8.804 kg, and those classified as low-yielding had an average yield of 5.216 kg (Table 5).

**Figure 2:**
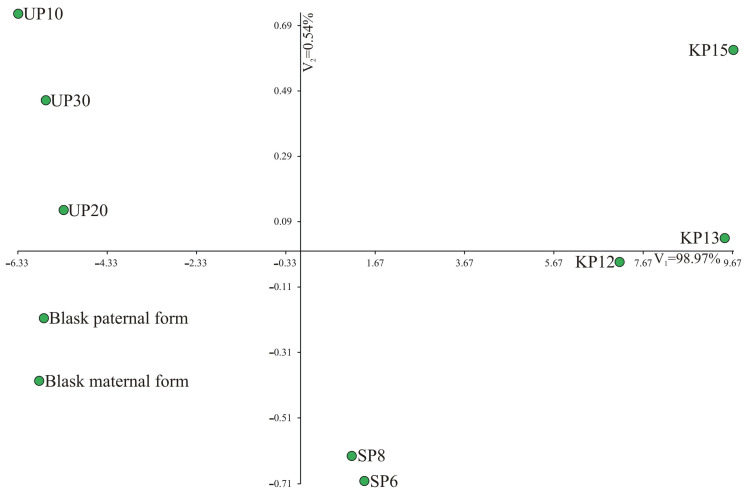
Distribution of the ten genotypes in the system of the first two canonical variates: V_1_ and V_2_.

The comparison of the yields of ten genotypes using multi-measure methods also confirmed a clear division into two groups: genotypes characterized by high yields and those characterized by low yields (Figure 2). A significantly greater amount of variability in multivariate genotype similarity/diversity is explained by the first canonical variability—98.97%. On the left side of the graph, there are low-yielding genotypes (UP10, UP20, UP30, Blask paternal form and maternal form), while on the right side, there are high-yielding genotypes (KP15, KP13, KP12, SP8 and SP6). Moreover, the genotypes are arranged according to their origin (on the left side above the X axis, there are genotypes from the PULS collection in Poznań—UP10, UP20, UP30; on the right side above the X axis and slightly below, there are genotypes from Małopolska Plant Breeding Co. Ltd.—KP12, KP13, KP15, while below the X axis, there are genotypes from Plant Breeding Smolice Co. Ltd. IHAR Group—Blask paternal form, Blask maternal form, SP8, SP6) (Figure 2).

### 3.2. Expression of Candidate Genes Linked to High Maize Yield

**Figure 3:**
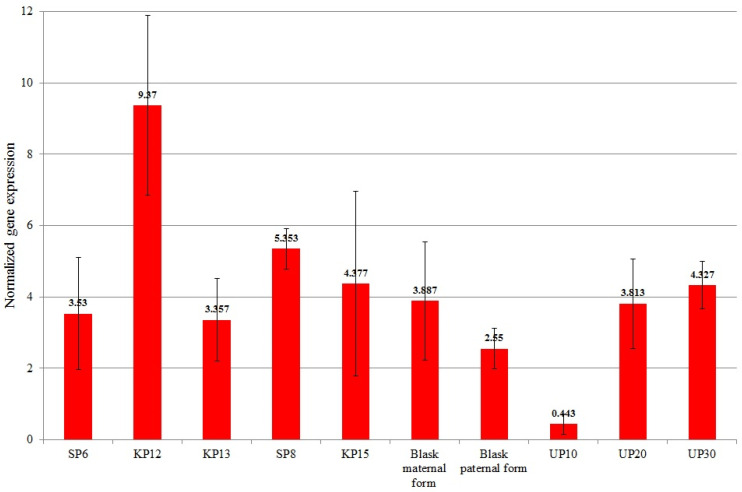
Mean values of normalized *Cinnamoyl-CoA reductase 1* (LOC103636116—1818 marker) gene expression for individual genotypes.

**Figure 4:**
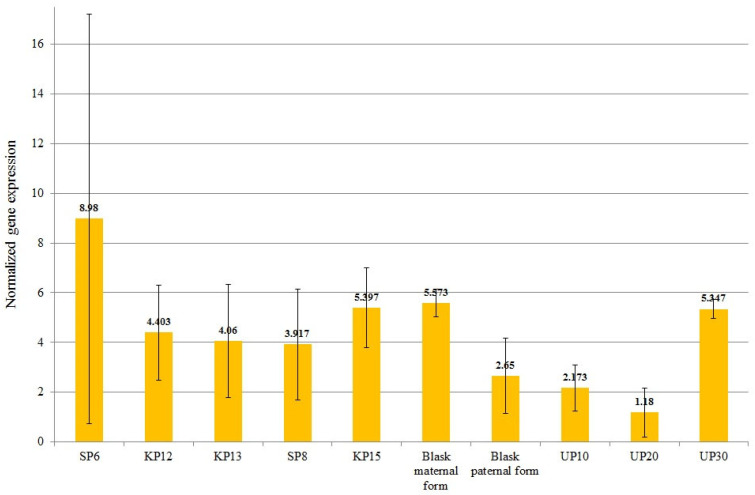
Mean values of normalized *WAT1-related protein At1g09380* (LOC103639851—14506 marker) gene expression for individual genotypes.

**Figure 5:**
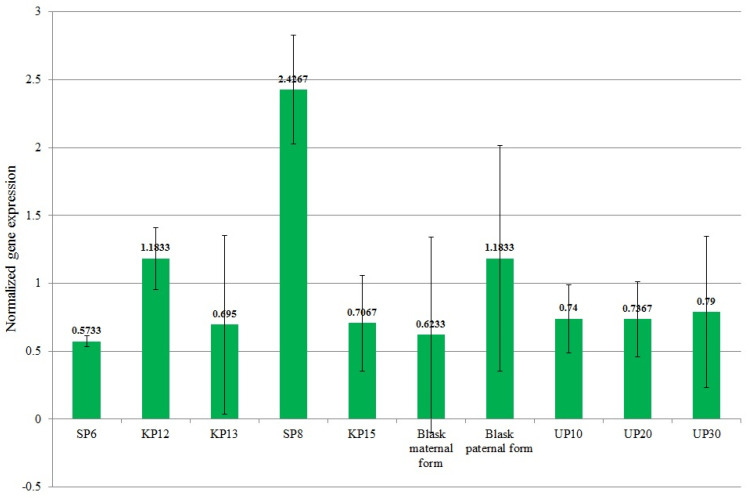
Mean values of normalized *Eukaryotic translation initiation factor 3 subunit c* (LOC100282883—16703 marker) gene expression for individual genotypes.

**Figure 6:**
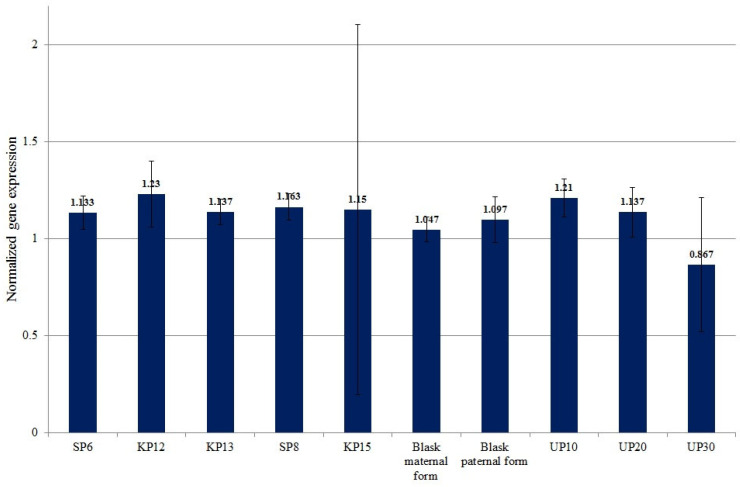
Mean values of normalized *RNA polymerase II transcriptional coactivator KELP* (LOC100285609—3233 marker) gene expression for individual genotypes.

**Figure 7:**
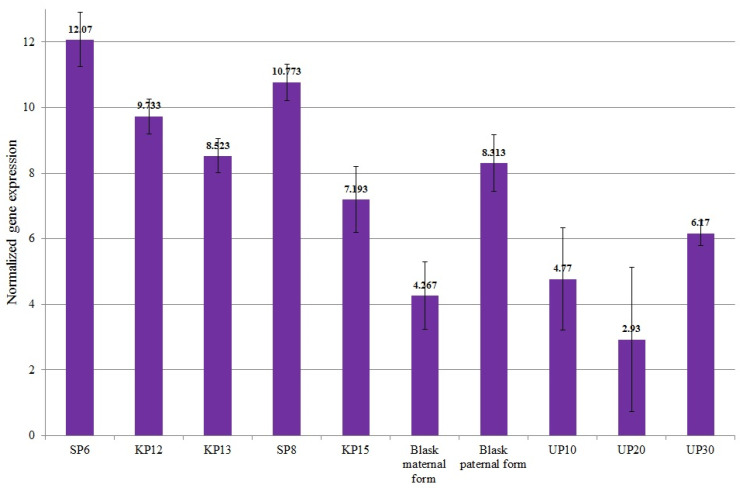
Mean values of normalized *Aspartate aminotransferase* (LOC100282625—11657 marker) gene expression for individual genotypes.

**Figure 8:**
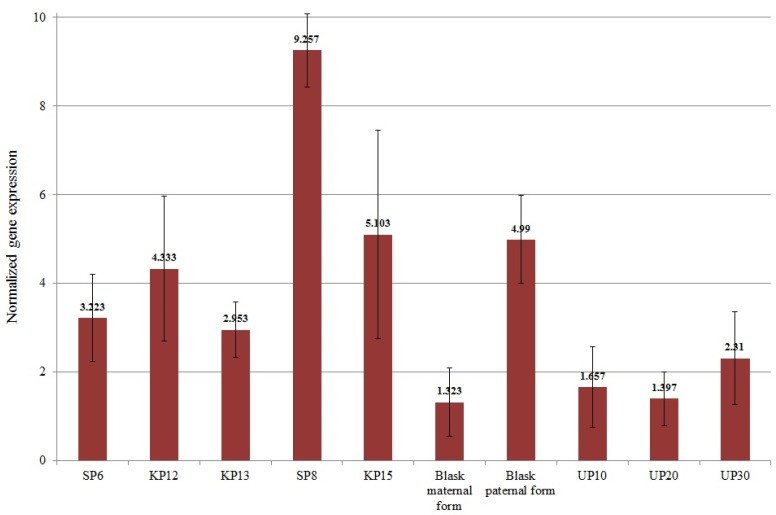
Mean values of normalized *Sucrose transporter 1* (LOC541615—12812 marker) gene expression for individual genotypes.

A one-way ANOVA revealed that the expression of all preselected genes (*KELP 0*) differed significantly between the studied genotypes (Table 7, Figure 3, Figure 4, Figure 5, Figure 6, Figure 7 and Figure 8).

A one-way ANOVA analysis revealed that the expression of *CCR1*, *AAT* and *SUT1* differed significantly between the studied groups, as determined by the yield (Table 8).

The mean normalized expression values of individual genes are presented in Figure 3, Figure 4, Figure 5, Figure 6, Figure 7 and Figure 8. The mean normalized expression of the *CCR1* gene ranged from 0.443 (for UP10—low-yielding) to 9.37 (for KP12—high-yielding). The mean expression value of the *CCR1* gene for high-yielding genotypes was 5.197 and was significantly higher than the mean expression value of low-yielding genotypes, which was 3.004 (Figure 3). The UP10 genotype, characterized by the lowest expression of the *CCR1* gene, was the lowest-yielding genotype in 2022–2024 (Figure 1).

The mean normalized expression of the *ArabidopsisWAT1* gene ranged from 1.18 (for UP20—low-yielding) to 8.98 (for SP6—high-yielding). The mean expression value of the *ArabidopsisWAT1* gene for high-yielding genotypes was 5.351 and was significantly greater than the mean expression value of low-yielding genotypes, which was 3.385 (Figure 4). The SP6 genotype, for which the highest expression of the *ArabidopsisWAT1* gene was recorded, was also characterized by the highest yield level in all years considered in the experiment (2022–2024) (Figure 1).

The mean normalized expression of the *EIF3C* gene ranged from 0.573 (for SP6—high-yielding) to 2.427 (for SP8—high-yielding). The mean expression value of the *EIF3C* gene for high-yielding genotypes was 1.117 and significantly exceeded the mean expression value of low-yielding genotypes, which was 0.814 (Figure 5). The SP8 genotype, which exhibited the highest expression of the *EIF3C* gene, was the second highest-yielding genotype in all of the years studied in the experiment (2022–2024) (Figure 1).

The mean normalized expression of the *KELP 0* gene ranged from 0.867 (for UP30—low-yielding) to 1.23 (for KP12—high-yielding). The mean expression value of the *KELP 0* gene for high-yielding genotypes was 1.162. The latter value was not significantly different from the mean expression value of low-yielding genotypes, which was 1.071 (Figure 6).

The mean normalized *AAT* gene expression ranged from 2.93 (for UP20—low-yielding) to 12.07 (for SP6—high-yielding). In high-yielding genotypes, the mean expression value of the *AAT* was 9.658, which significantly exceeded the 5.29 expression value observed in low-yielding genotypes (Figure 7). The SP6 genotype, for which the highest expression of the *AAT* gene was recorded, was also characterized by the highest yield in all the studied years (2022–2024) (Figure 1).

The mean normalized expression of the *SUT1* gene ranged from 1.323 (for Blask maternal form—low-yielding) to 9.257 (for SP8—high-yielding). The mean expression value of the *SUT1 1* gene for high-yielding genotypes was 4.973 and was significantly greater than the mean expression value of low-yielding genotypes, which was 2.335 (Figure 8). The SP8 genotype, which exhibited the strongest expression level of the *SUT1 1* gene, was the second highest-yielding genotype in all the studied years (2022–2024) (Figure 1).

**Figure 9:**
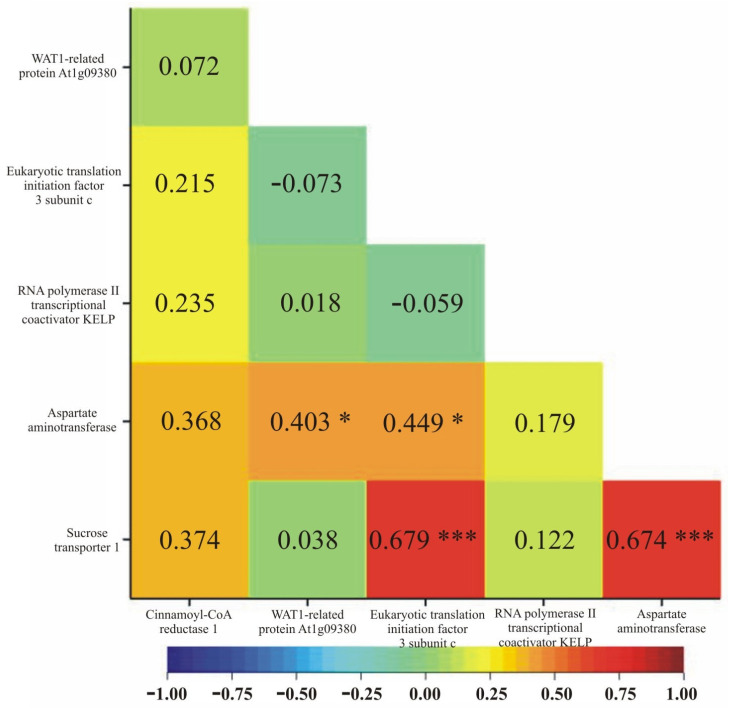
Linear correlation coefficients for normalized gene expression between pairs of six genes. * *p* < 0.05; *** *p* < 0.001.

The correlation of normalized gene expression between individual genes was estimated based on Pearson’s linear correlation coefficients. This analysis revealed that some genes exhibited similar expression distributions across individual genotypes. Statistically significant correlations in normalized expression were found between the following gene pairs: *AAT* and *ArabidopsisWAT1* (0.403); *AAT* and *EIF3C* (0.449); *SUT1* and *EIF3C* (0.679); as well as *SUT1* and *AAT* (0.674) (Figure 9).

**Figure 10:**
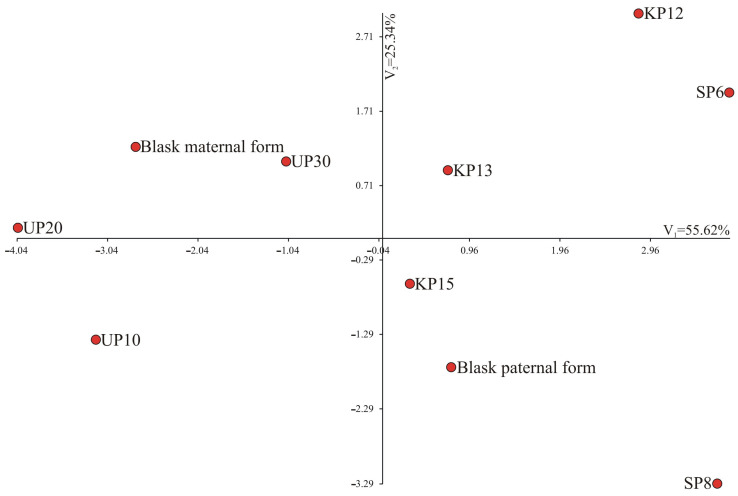
Distribution of the ten genotypes in the system of the first two canonical variates: V_1_ and V_2_.

A comparison of the normalized gene expression of ten genotypes using multivariate methods is presented in Figure 10. Canonical variate analysis was performed to check whether the analyzed genotypes were grouped depending on the expression level of individual genes. On the left side of the Y axis, low-yielding genotypes are clustered and characterized by low expression levels of these genes. Conversely, on the right side of the Y axis, high-yielding genotypes are grouped, showing high expression levels of the analyzed genes. The first canonical variate explained 55.62% of the total genotype variability, whereas the second canonical variate determined the genotype variability in 25.34% (Figure 10). The greatest multidimensional similarity in terms of normalized expression of six genes, by the Mahalanobis distance, was observed for Blask maternal form and UP30 (1.853). The most diverse genotypes were SP6 and UP20 (9.02) (Table 9). The greatest multidimensional similarity in terms of yield, expressed by the Mahalanobis distance, was observed for Blask maternal form and Blask paternal form (0.283). The most diverse genotypes were KP15 and UP10 (16.018) (Table 9).

### 3.3. The Effect of Normalized Gene Expression on Yield in Years

Two genes, *CCR1* and *AAT*, out of the six studied significantly influenced the yield of the evaluated genotypes over the three years of observation. The percentage of yield variability explained by the normalized expression of the *CCR1* gene was 11.40% in 2022, 15.30% in 2023, and 15.60% in 2024. For the *AAT* gene, the percentages were: 23.10% in 2022, 13.20% in 2023 and 22.80% in 2024 (Table 10). Additionally, the *SUT1* gene also had a significant impact on maize yield, with an effect size of 0.33, explaining 10.00% of the yield variability among the genotypes (Table 10).

### 3.4. Transcriptomic Data Analysis

**Figure 11:**
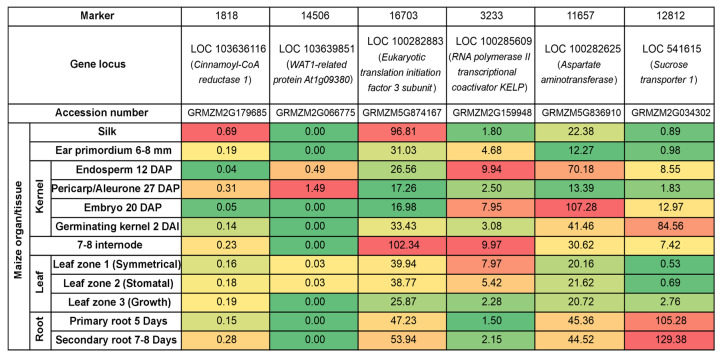
A heatmap showing changes in the expression levels of five candidate genes within which significant SilicoDArT markers are located in selected *Zea mays* organs and tissues. Locus and accession numbers that enabled analysis of transcriptomic data are presented. The heatmap depicts Fragments Per Kilobase per Million mapped fragments (FPKM) values in transcriptomic data acquired from Walley and collaborators [33] For each gene (columns), red indicates the highest, green indicates the lowest, and yellow-orange indicates moderate gene expression. DAP—days after pollination. DAI —days after initiation.

The expression level of identified genes in various organs and tissues of *Z. mays* was analyzed with the use of public available transcriptomic data [33]. Gene LOC103636116, harboring marker 1818, was most strongly expressed in silk. Transcript levels of four of the genes, namely LOC103639851 (14506 marker), LOC100285609 (3233 marker), LOC100282625 (11657 marker), and LOC541615 (12812 marker) were found to be increased in various compartments of kernels, i.e., in the pericarp/aleurone, endosperm, embryo, and in germinating kernels. Expression of LOC100285609 (3233 marker) was also increased in the internode and symmetrical leaf zone, while LOC541615 (12812 marker) was expressed predominantly in the root. Gene LOC100282883 (16703 marker) was expressed mainly in internode and silk (Figure 11).

### 3.5. Identification of SilicoDArT Markers Linked to Candidate Genes Determining Maize Grain Yield

**Figure 12:**
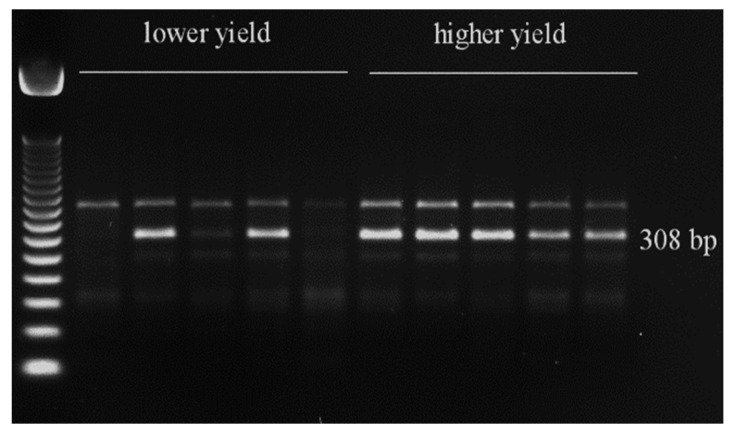
PCR profiling of low and high-yielding maize genotypes. The marked 308 bp amplification product is specific to SNP marker 3233, which is linked to *RNA polymerase II transcriptional coactivator KELP*.

**Figure 13:**
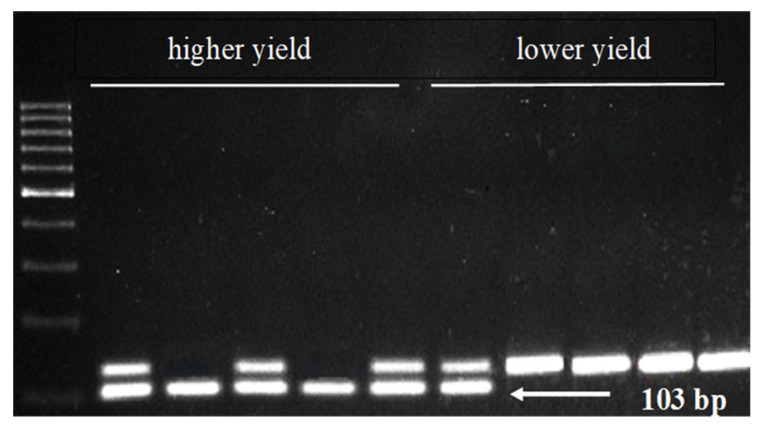
PCR profiling of low- and high-yielding maize genotypes. The marked 103 bp amplification product is specific to SNP marker 11657, which is linked *to the Aspartate aminotransferase* gene.

Using polymerase chain reaction (PCR), the selected markers were tested on 10 reference genotypes with high and low yield. Of the six markers tested, only two (3233 and its associated gene—*KELP 0* and 11657 and its associated gene—*AAT*) distinguished between low and high yielders (Figure 12 and Figure 13). In the case of marker 3233, a 308 bp product indicating the presence of the *KELP 0* gene occurred in all high yielders and two low yielders. In the case of marker 11657, a 103 bp product indicating the presence of the *AAT* gene occurred in all high yielders and one low yielder (Figure 12 and Figure 13).

## 4. Discussion

The ever-increasing competition in the food market requires breeders and food producers to have access to the most advanced molecular techniques that can be used to create biological progress. This progress, in turn, will contribute to the creation of new, productive crop varieties that can be grown in different climatic zones, including regions where planting was previously deemed unprofitable [34]. Understanding the genetic structure of the various crop species comprising maize has become a priority for many scientists [35]. Such a genomics-oriented approach allows for all the necessary information about coding regions that provide insights into gene (protein) structure, as well as details regarding inter-gene regions, to be obtained [36]. Moreover, with the advancement of high-throughput DNA sequencing methods, which enable the determination of whole-genome and -transcriptome sequences, a new quality of research has emerged in many plant species, including maize [37,38]. Since the sequencing of the first model plant genome in 2000, sequences of over 100 other plant species have been recorded [39,40].

In recent years, many authors have tried to identify molecular markers that are functionally related to important maize traits. Tomkowiak [41] attempted to identify molecular markers linked to genes that showed resistance to maize smut. Bocianowski et al. [42] used next-generation sequencing and association mapping to identify markers linked to the heterosis effect in maize. Using the same methods, Sobiech et al. [43] identified markers associated with maize plant resistance to fusarium. NGS technology is used to sequence genomes and transcriptomes, study protein DNA/RNA interactions, assess methylation levels, discover new DNA polymorphisms, and conduct metagenomic studies [44]. Additionally, it allows for the analysis of different DNA fragments represented by multiple copies in a single reaction, library preparation, and then collection of gigabases of genomic data from a single sequencing run [45,46]. This approach increases both the number of analyzed samples and the reliability of sequencing results, which is particularly useful when differences between specific genotypes are minimal [36]. Next-generation sequencing techniques also enable qualitative and quantitative analyses of genes expressed under different conditions, and the results of these analyses are used for association mapping [47,48,49,50].

In the previous study, DArTseq technology, utilizing the NGS platform, combined with association and physical mapping, was used to identify molecular markers and linked candidate genes that determine maize yield [26]. Six significant SNP molecular markers, namely, 1818, 14606, 16703, 3233, 11657, and 12812, were preselected [26]. These markers are located on chromosomes 8, 9, 7, 3, 5, and 1, within: *CCR1, ArabidopsisWATA, EIF3C, KELP 0, AAT, SUT1* genes, respectively. In this publication, an attempt was made to characterize the aforementioned candidate genes and evaluate their role in the formation of maize yield. In addition, their expression was analyzed in reference materials with high and low yields.

Over a three-year period (2022, 2023, and 2024), we conducted a field experiment using reference genotypes to estimate their yields. The results indicated that maize yield followed a normal distribution. The genotypes identified as high-yielding averaged 8.804 kg, while those classified as low-yielding had an average yield of 5.216 kg. The results of the two-factor analysis of variance showed that genotypes and years were factors that significantly differentiated the yield. In 2024, both high-yield and low-yield genotypes achieved their maximum yields. An analysis of the weather data for 2024 revealed that the highest average temperature and rainfall were recorded in this year. Since maize is a thermophilic C4 plant, it requires higher temperatures than many other crops for optimal growth. As reported by other authors, grain composition traits are strongly interrelated and also respond to weather conditions [51,52,53]. It is also known that the yield and quality of maize grain are the result of interactions between genetic, environmental and agronomic factors. Although genotype has a large impact on grain composition [54], temperature and humidity during key physiological periods of growth play the greatest role.

In the current study, the yield of the analyzed genotypes was correlated with the expression level of candidate genes, within which significant molecular markers were located. The first gene whose expression was analyzed was *CCR1*, within which marker number 1818 was located. According to reports from the literature, *CCR1* is considered to be a key enzyme that controls the quantity and quality of lignin. The first transgenic plants with reduced *CCR1* activity were obtained for tobacco. The lignin content was reduced by 50% compared to the amount of lignin in the wild-type. However, the decrease in lignin content had a detrimental effect on the development of tobacco plants. *CCR1* biosynthesis was characterized in detail in dicotyledonous plants. Unfortunately, there are still only a few reports available on monocotyledonous plants [55]. Our studies indicated that the *ZmCCR1* gene had a significant influence on maize yield over three consecutive years of experimentation. Research by López-Malvar [56] demonstrated that a high proportion of lignin subunit G, coupled with a low concentration of p-coumaric acid and lignin subunit S, contributes positively to rind puncture resistance and results in taller plants, ultimately increasing biomass yield. Additionally, a greater proportion of subunit H is associated with longer internodes. Furthermore, lower hemicellulose content correlates with enhanced rind puncture resistance. These findings confirm the impact of the cell wall on agronomic and stalk traits, which could be valuable for applied breeding programs aimed at improving biomass yield. According to Zhang et al. [57], lignin occurs in the cell wall and is essential for the transport of water and nutrients in plant stems. While the polysaccharide components of plant cell walls are hydrophilic and allow for water permeability, lignin is hydrophobic. The cross-linking of polysaccharides by lignin restricts water absorption by the cell wall. This characteristic enables the conductive tissue of the plant to efficiently transport water, which is vital for the plant’s proper functioning, growth, and development, ultimately influencing its yield. In view of the above, it is highly likely that the *CCR1* gene is associated with yield height.

Since protein synthesis during the grain filling phase relies on amino acid remobilization from vegetative tissues, amino acid transporters are expected to be involved in this process [58]. Amino acid translocation is thought to be one of the important factors determining seed quality traits. In this study, a correlation between maize yield and the presence of molecular marker 14506, located inside the *ArabidopsisWAT1* gene (LOC103639851), has been demonstrated. *ZmWAT1-related* protein is a close homolog of the *UMAMIT25 (At1g09380*) from *Arabidopsis thaliana*, which belongs to the Usually Multiple Amino acids Move In and Out Transporter (*UMAMIT*) family. Members of *UMAMIT* constitute bidirectional amino acid transporters, as well as vacuolar auxin transporters involved in the formation of the secondary cell wall in *Arabidopsis* [59,60]. The *AtUMAMIT25* is localized at the plasma membrane of endosperm and pericarp cells, which suggests its role in amino acid export from the endosperm and/or pericarp to the developing seeds. In comparison to wild-type plants, *umamit25* knockout lines exhibit reduced amino acid content during embryogenesis. Additionally, an increment in *AtUMAMIT25* expression results in an increase in seed number and mass. Notably, similar tissue expression patterns in the endosperm and pericarp of the kernel have been described for the *WAT1-related* gene in maize (Figure 11). Based on gene homology, expression analyses (Figure 11), and observed differences in its level within high- and low-yielding genotypes (Table 7, Figure 4), *ZmWAT-1* might be one of the attractive candidate genes, presumably mediating yield.

*EIF3C* are among the most important components in plant protein synthesis [61]. Over the past 20 years, several plant *EIF3C* subunits have been identified and analyzed, mainly using *Arabidopsis thaliana*, but their direct association with yield is still missing [62]. In this study, the molecular marker SNP 2317 was found to be localized within the gene encoding *EIF3C*. The expression of the latter differed significantly between the studied maize genotypes (Table 7). However, the influence of its expression on the genotype yields in the individual years was not statistically significant (Table 10). In *Arabidopsis*, the expression pattern of *EIF3C* genes is primarily observed in actively proliferating tissues, such as apical meristems, leaf primordia, inflorescences, developing anthers, and germinating seeds, which substantially require translational machinery. [62]. In maize, the *EIF3C* gene is expressed mainly in 7–8 internodes and silk (Figure 11), which might suggest its role in growth and reproduction, respectively.

The fourth SNP marker, 3233, is located within the *KELP 0* gene. In eukaryotes, *RNA polymerase II* is essential for transcribing protein-coding genes and small non-coding RNAs, while coactivators are crucial elements of the RNA polymerase II complex [63]. In *Arabidopsis thaliana*, *AtKELP/AtNMDM2* has the ability to bind to tomato mosaic virus (*ToMV*) and can interfere with virus movement into the cell, thus participating in the immune response [64]. Authors have associated this gene with the immune response to abiotic stress caused by high temperatures. Additionally, it has been shown that *AtKELP/AtNMDM2* interacts with *AtNMDM1* in pollen nuclei. Both proteins are positive regulators of pollen intine formation, thereby influencing male fertility. Dysfunction of *AtNMDM1* downregulates the expression of intine-related genes, resulting in abnormal cellulose distribution within the intine and causing pollen defects. In our studies, no significant expression of gene encoding putative *KELP* coactivator was observed in the analyzed genotypes (Table 9, Figure 6). However, the marker associated with this gene (3233) differentiated high- and low-yielding genotypes on agarose gels (Figure 12). Moreover, mRNA of *ZmKELP* accumulated strongly in endosperm and embryos (Figure 11). In view of the above, its relationship with yield should be reanalyzed.

The fifth significant SNP marker, 11657, is anchored inside the gene encoding *AAT*. The latter is a crucial enzyme involved in amino acid synthesis. It plays a significant role in regulating carbon and nitrogen metabolism, especially in C4 plant species [65]. It has been shown that overexpression of *AAT* genes in rice resulted in greater seed amino acid and protein contents [66]. A significant effect of *ZmAAT* gene expression on yield was demonstrated in three consecutive years of the experiment (Table 10). Moreover, the *ZmAAT* transcript was found to be present in all analyzed maize tissues with the highest expression level in the kernel, mainly in the embryo and endosperm (Figure 11). In addition, the marker linked to this gene differentiated high- and low-yielding genotypes (Figure 13). The obtained results allow us to conclude that the *ZmAAT* gene (LOC100282625) is associated with yield in maize.

Transport of photoassimilates from the source leaves to different sink tissues (e.g., ears, tassels, stems, and roots) is essential to plant growth, development, and thus crop yield [67]. Sucrose is the principal form of carbohydrate that is translocated over long distances [68,69]. Notably, one of the described SNP markers associated with a higher-yield phenotype, namely 12812, is anchored inside the sucrose transporter 1 gene (LOC541615). *ZmSUT1* belongs to the sucrose transporter family (*SUTs/SUCs*) [70]. It constitutes a plasma membrane-localized protein, participating in sucrose loading in phloem companion cells as well as its retrieval from the apoplast [70]. Disruption in *ZmSUT1* resulted in hyperaccumulation of carbohydrates in mature leaves. As a consequence, knockout lines exhibit vegetative growth defects with reduced total biomass, as well as stunted reproductive development with arrested tassel and ears formation and delayed flowering. Moreover, reduction in chlorophyll accumulation, photosynthesis and stomatal conductance has also been observed in mutant lines [71]. Analysis of publicly available transcriptomic data revealed that *ZmSUT1* is ubiquitously expressed in both assimilating organs and various sink tissues, with the strongest mRNA accumulation in roots and germinating kernel (Figure 11). A similar observation was made by Baker et al. [67] and is consistent with the significant effect of *ZmSUT1* on yields.

In addition to verifying the expression levels of selected genes in different maize genotypes, the question of whether their expression influenced yield was addressed. Moreover, using the Pearson coefficient method, the linear correlation of gene expression between individual genes was estimated (Figure 9). The expression of the *AAT*- and *WAT1*-related, *AAT* and *eIF3c*, *AAT* and *SUT1*, and *SUT1* and *eIF3c* gene pairs showed similar distribution in individual genotypes. Three genes, *AAT*, *SUT1*, and *eIF3c,* are predominantly present in correlating pairs. Interestingly, genes that are putatively responsible for amino acid formation (*AAT*) and translocation (*WAT1-related*) correlated with each other. Similar relationships were observed in the studies of Bociankowski et al. [72,73].

## 5. Conclusions

A detailed analysis of selected candidate genes allowed us to identify three out of six genes—CCR1, AAT, SUT1 that can significantly influence grain yield in maize. Our studies, along with literature reports, clearly demonstrate the roles of these genes in yield formation. The molecular markers identified within their sequence can be utilized in breeding programs to select high-yielding maize genotypes. Additionally, the application of modern biotechnological tools can accelerate the development of new varieties, thereby contributing to global food security.

## Figures and Tables

**Table 1:** Selected yield-related candidate genes and their associated SNP molecular markers.

Marker	Marker Type	Chromosome	Marker Location	Associated with	Candidate Genes
1818	DArT	Chr8	1.5 × 10^8^	Cob diameter, the number of rows of grain, mass of grain from the cob, yield	A marker that is anchored to the gene *cinnamoyl-CoA reductase 1*
14506	DArT	Chr9	28978769	Cob diameter, the number of rows of grain, mass of grain from the cob, yield	A marker that is anchored (*WAT1-related protein At1g09380*)
2317	DArT	Chr7	1.38 × 10^8^	Cob diameter, the number of rows of grain, mass of grain from the cob, yield	A marker that is anchored (*eukaryotic translation initiation factor 3 subunit c*)
3233	DArT	Chr3	2.1 × 10^8^	Cob diameter, the number of rows of grain, mass of grain from the cob, yield	A marker that is anchored *RNA polymerase II transcriptional coactivator KELP*
11657	DArT	Chr5	2.22 × 10^8^	Cob diameter, core diameter, mass of grain from the cob, yield	A marker that is anchored *aspartate aminotransferase*
12812	DArT	Chr1	15198950	Cob length, core length, the number of rows of grain, weight of one thousand grains	A marker that is anchored *sucrose transporter 1*

**Table 2:** Sequences and annealing temperatures (Ta) of primers used in qPCR.

Candidate Gene and Its Linked SNP Markers	Forward and Reverse Primer (5′→3′)		Annealing Temperature (Ta)	Product Size (bp)
*Cinnamoyl-CoA reductase 1* (1818 marker)	GGAGGCAGGACTACCCTCAT	TTTTGCCAAGTGCGAACCAC	60	111
*WAT1-related protein At1g09380* (14506 marker)	AAAGGTGGCGCTACTTACCC	AGAAGCTTTGCAAATTGAGCTT	57	110
*Eukaryotic translation initiation factor 3 subunit c* (2317 marker)	CTCTACTCTACTGGGAGGGTG	GGCATTTGCCTTCTCCTCTCA	58	110
*RNA polymerase II transcriptional coactivator KELP* (3233 marker)	GTTCTACGTGAAGGACGGCA	TCTATCGCAGGTGCAGCATT	59	99
*Aspartate aminotransferase* (11657 marker)	GGAGGAGTTAACCAGGCGAG	AGCCATTGGCACTCCTTCAA	59	123
*Sucrose transporter 1* (12812 marker)	CGCCTCCCAAAGCTCTCTT	GACCCCCACGGACAGCTC	59	124
*β tubulin* (β-TUB)	CTACCTCACGGCATCTGCTATGT	GTCACACACACTCGACTTCACG	61	139
*Cyclophilin* (CYP)	CTGAGTGGTGGTCTTAGT	AACACGAATCAAGCAGAG	59	100

**Table 3:** Sequences and annealing temperatures (Ta) of primers used in PCR.

Marker	Forward and Reverse Primer (5′→3′)	Annealing Temperature (T_a_)	Product Size (bp)
14506	ACACTGGGAGAGGAGGAGG	57	217
	TACTATTATATTCTCCCATACATGC		
16703	CCATCGTGATCTCCAAACAGCG	60	367
	GCTGAAGTTACACGTACCGTAAG		
3233	AGCAATACCTTGATTCTGTTATGCC	58	308
	TTCTTTAAACCAAACCAAATTTCTC		
11657	GAAGCCCATCAGAGGCATGTCTTATT	58	103
	AAGCCTATGCCAGCTAGGTATTT		
12812	AAGGTAAAAAGCTATATATATATGA	60	219
	AACAGCAACCAAAAGTCAC		

**Table 4:** Results of a two-way analysis of variance for yield.

Source of Variation	The Number of Degrees of Freedom	Sum of Squares	Mean Square	*F*-Statistic
Genotype	9	358.08	39.79	116.44 ***
Year	2	13.67	6.83	20.00 ***
Genotype × Year	18	6.00	0.33	0.98 ^ns^
Residual	60	20.50	0.34	
Total	89	398.26		

*** *p* < 0.001; ns—not-significant.

**Table 5:** Mean values and standard deviations of yields for genotypes in individual years (interactive means), genotypes by years, and a priori determined groups of high- and low-yielding genotypes.

Year	2022		2023		2024		Average	
Genotype	Mean	s.d.	Mean	s.d.	Mean	s.d.	Mean	s.d.
SP6	7.033	0.2838	6.833	0.6611	8.237	0.8113	7.368 c	0.8521
KP12	8.497	0.3866	9.257	0.2458	10.407	0.5331	9.387 b	0.9039
KP13	9.7	0.5203	9.373	0.8186	10.48	0.5672	9.851 ab	0.7471
SP8	6.953	0.3525	6.797	0.5008	8.13	0.1277	7.293 c	0.7044
KP15	9.573	0.5607	10.047	0.5372	10.747	0.4262	10.122 a	0.6764
Blask maternal form	4.92	0.1389	4.833	0.5862	5.71	0.4636	5.154 d	0.5652
Blask paternal form	5.037	0.0737	4.887	0.5972	5.607	0.3968	5.177 d	0.488
UP10	5.063	0.6987	5.1	0.8731	5.05	0.943	5.071 d	0.7317
UP20	5.04	0.6678	5.4	1.0173	5.903	0.7537	5.448 d	0.8082
UP30	5.213	0.3083	5.147	0.1557	5.33	0.8585	5.23 d	0.4696
Average	6.703 B	1.918	6.767 B	2.062	7.56 A	2.308		
High-yielding genotypes	8.351	1.2804	8.461	1.5026	9.6	1.2874	8.804 G1	1.447
Low-yielding genotypes	5.055	0.3999	5.073	0.6358	5.52	0.6801	5.216 G2	0.612

a, b, c, d—individual letters denote different homogeneous groups. The means and overall years of observation are also stated. Genotypes grouped in one group do not differ statistically; A, B denote two homogeneous groups for the years of management by genotypes; G1 and G2 denote different homogeneous groups for genotypes classified as high- and low-yielding.

**Table 6:** Results of a one-way analysis of variance for yield determined by high- and low-yielding genotype groups.

Source of Variation	The Number of Degrees of Freedom	Sum of Squares	Mean Square	*F*-Statistic
Group	1	289.695	289.695	264.10 ***
Year	2	13.669	6.835	6.23 **
Group × Year	2	2.752	1.376	1.25 ^ns^
Residual	84	92.142	1.097	
Total	89	398.258		

** *p* < 0.01; *** *p* < 0.001; ns—not significant.

**Table 7:** Mean squares from a one-way analysis of variance for normalized expression of six genes.

Source of Variation	Genotype	Residual
The number of degrees of freedom	9	20
*Cinnamoyl-CoA reductase 1*	15.417 ***	2.226
*WAT1-related protein At1g09380*	14.324 *	5.802
*Eukaryotic translation initiation factor 3 subunit*	0.925 **	0.2323
*RNA polymerase II transcriptional coactivator KELP*	0.0312 ^ns^	0.1116
*Aspartate aminotransferase*	26.315 ***	1.224
*Sucrose transporter 1*	17.579 ***	1.409

* *p* < 0.05; ** *p* < 0.01; *** *p* < 0.001; ns—not significant.

**Table 8:** Mean squares from a one-way analysis of variance for normalized expression of six genes determined by yield groups.

Source of Variation	Group	Residual
The number of degrees of freedom	1	28
*Cinnamoyl-CoA reductase 1*	36.08 *	5.257
*WAT1-related protein At1g09380*	23.946 ^ns^	7.273
*Eukaryotic translation initiation factor 3 subunit*	0.8291 ^ns^	0.4393
*RNA polymerase II transcriptional coactivator KELP*	0.06256 ^ns^	0.08753
*Aspartate aminotransferase*	147.288 ***	4.301
*Sucrose transporter 1*	52.219 **	4.792

* *p* < 0.05; ** *p* < 0.01; *** *p* < 0.001; ns—not-significant.

**Table 9:** Mahalanobis distances between analyzed genotypes for yield (upper diagonal) and normalized gene expression (lower diagonal).

Genotype	SP6	KP12	KP13	SP8	KP15	Blask Maternal Form	Blask Paternal Form	UP10	UP20	UP30
SP6	0	5.853	8.134	0.295	8.372	7.291	7.194	7.882	6.817	7.226
KP12	4.908	0	2.945	6.114	2.741	13.029	12.934	13.534	12.455	12.91
KP13	3.696	4.483	0	8.411	1.164	15.385	15.266	15.849	14.866	15.218
SP8	6.874	6.441	6.153	0	8.638	7.005	6.907	7.591	6.525	6.936
KP15	5.395	5.128	3.123	4.894	0	15.577	15.464	16.018	15.008	15.4
Blask maternal form	7.457	6.169	4.454	7.965	3.781	0	0.283	1.246	0.887	0.932
Blask paternal form	5.28	5.832	2.765	4.28	2.398	5.057	0	1.098	0.847	0.685
UP10	7.949	8.059	4.535	7.764	4.348	3.623	4.022	0	1.388	0.677
UP20	9.02	7.473	5.541	8.511	4.85	2.265	5.53	3.048	0	0.947
UP30	6.078	4.741	3.175	6.534	2.914	1.853	3.889	3.929	3.391	0

**Table 10:** The impact of normalized gene expression on yield of studied genotypes over the years.

Year	Gene	Effect	Standard Error	*p*-Value	Percentage Variance Accounted for
2022	*Cinnamoyl-CoA reductase 1*	0.29	0.133	0.038 *	11.40
	*WAT1-related protein At1g09380*	0.133	0.13	0.317	0.10
	*Eukaryotic translation initiation factor 3 subunit c*	0.171	0.533	0.751	x
	*RNA polymerase II transcriptional coactivator KELP*	0.6	1.23	0.626	x
	*Aspartate aminotransferase*	0.322	0.105	0.005 **	23.10
	*Sucrose transporter 1*	0.259	0.134	0.064	8.60
2023	*Cinnamoyl-CoA reductase 1*	0.35	0.14	0.019 *	15.30
	*WAT1-related protein At1g09380*	0.108	0.142	0.456	x
	*Eukaryotic translation initiation factor 3 subunit c*	−0.077	0.579	0.896	x
	*RNA polymerase II transcriptional coactivator KELP*	1.32	1.3	0.320	0.10
	*Aspartate aminotransferase*	0.276	0.121	0.030 *	13.20
	*Sucrose transporter 1*	0.255	0.146	0.091	6.60
2024	*Cinnamoyl-CoA reductase 1*	0.395	0.157	0.018 *	15.60
	*WAT1-related protein At1g09380*	0.236	0.157	0.145	4.50
	*Eukaryotic translation initiation factor 3 subunit c*	0.308	0.656	0.642	x
	*RNA polymerase II transcriptional coactivator KELP*	0.67	1.48	0.654	x
	*Aspartate aminotransferase*	0.387	0.127	0.005 **	22.80
	*Sucrose transporter 1*	0.33	0.16	0.049 *	10.00

* *p* < 0.05; ** *p* < 0.01; x—residual variance exceeds variance of response variate.

## Data Availability

The data presented in this study are available on request from the corresponding author as the data also form part of an ongoing study.
